# An unusual case of remission of clinically overt autoimmune Addison’s disease in a Pakistani girl

**DOI:** 10.12669/pjms.41.8.11649

**Published:** 2025-08

**Authors:** Kashan Arshad, Syed Saddam Hussain, Sommayya Aftab

**Affiliations:** 1 Kashan Arshad Department of Pediatric Endocrinology and Diabetes, University of Child Health Sciences, The Children’s Hospital, Lahore, Pakistan; 2 Syed Saddam Hussain Department of Pediatric Endocrinology and Diabetes, University of Child Health Sciences, The Children’s Hospital, Lahore, Pakistan; 3 Sommayya Aftab Department of Pediatric Endocrinology and Diabetes, University of Child Health Sciences, The Children’s Hospital, Lahore, Pakistan

**Keywords:** Addison disease, Celiac disease, Recovery

## Abstract

Addison’s disease is generally regarded as an autoimmune irreversible, progressive disease leading to the lifelong replacement of glucocorticoids and mineralocorticoids. We are reporting a rare case of a young girl diagnosed with clinically overt Addison’s disease whose adrenal function recovered over time. A 10.5 years old girl, known case of celiac disease for five years with good compliance presented at 7.5 years with complaints of dark pigmentation, lethargy, nausea, and vomiting. Physical examination showed hyperpigmentation along with orthostatic hypotension. Biochemical tests confirmed Addison’s disease, with normal electrolytes, normal renin and raised ACTH. She was prescribed hydrocortisone tablets and showed significant improvement.

She was lost to follow up for three years and presented again with history that she had stopped hydrocortisone 1.5 years back and doing well. She was found clinically stable with no pigmentation, normal growth parameters and normal recent serum electrolytes. Her synacthen testing showed basal ACTH of 282 pg/mL, 30 minutes serum cortisol of 500 nmol/L, and 60 minutes cortisol of 576.63 nmol/L. In her last visit (11 years of age), she was thriving well, with an early morning ACTH of 126 pg/mL and a cortisol level of 510 nmol/L. This is the first case in children with clinical remission of autoimmune Addison’s disease. We suggest re-assessing the adrenal axis by synacthen test in children and adolescents with Addison disease, especially those whose hydrocortisone doses are gradually decreasing, to check for remission.

## INTRODUCTION

Addison’s disease (AD) is generally regarded as an irreversible autoimmune disease leading to the lifelong replacement of glucocorticoids and mineralocorticoids.^1^ AD and Celiac disease (CD) may coexist, although this is uncommon in youngsters.^2^ The best way to diagnose Addison’s disease is with a short synacthen test (250 µg). If this test is not readily available, an initial screening method involving the measurement of morning plasma adrenocorticotropic hormone (ACTH) and cortisol levels should be considered. We are reporting a rare case of a patient diagnosed with clinically overt Addison’s disease who showed recovery of adrenal functions over time. This appears to be the first ever recorded case of spontaneous remission in children.

## CASE PRESENTATION

A 10.5 years old female presented at the age of five years with a history of off-and-on abdominal pain and loose motion for the last six months. At that time, the patient was diagnosed with celiac disease on the basis of positive anti-tissue transglutaminase IgA and IgG antibodies and remained on a gluten-free diet with good compliance. At 7.5 years of age, she exhibited symptoms of lethargy, nausea, and vomiting associated with a darkening of color. There was no relevant family history. Physical examination revealed dark pigmentation of the skin and gingiva with orthostatic hypotension, a weight of 26 kg (at the 75th centile), and a height of 123 cm (at the 50th centile). Baseline biochemistry was highly suggestive of Addison’s disease: sodium 142 mEq/L, potassium 5.7 mEq/L, basal cortisol (8 a.m.) 45.79 nmol/L (normal 140 to 690 nmol/L), ACTH 1250 pg/ml (normal <= 46.0 pg/mL), and serum renin 3.9 uIU/mL (normal 2.8 to 39.9 uIU/mL). Thyroid function was normal, and adrenal antibodies were not checked due to their non-availability in Pakistan. Patient was started on tab. hydrocortisone (12 mg/m2/day) eight hourly along with sick day management education. The patient was lost during follow up for three years and presented back at 10 years of an age with history of self-stopping of hydrocortisone since last 1.5 year and doing well.

She was thriving well with a height of 140 cm (at the 50^th^ centile), a weight of 50 kg (> 90^th^ centile), and no pigmentation despite being off hydrocortisone. Investigations revealed normal serum electrolytes, normal serum renin (39.2 uIU/ml), and early morning cortisol of 408.33 nmol/L. Her synacthen testing showed basal ACTH of 282 pg/mL, 30 min serum cortisol of 500 nmol/L, and 60 minutes cortisol of 576.63 nmol/L. The patient was not started on hydrocortisone and was kept on regular follow-up. In her last visit (11 years of age), she was thriving well, with an early morning ACTH of 126 pg/mL and a cortisol level of 510 nmol/L.

## DISCUSSION

Celiac disease is a condition leading to a variety of important nutrient deficits. Since many of these nutrients are required for healthy adrenal function, they may have an impact on adrenal function.^3^ A link between AD and CD has already been recognized, although there have been very few documented pediatric cases.^4^ The positive correlation between CD and AD, observed both before and after CD diagnosis, may be explained by the presence of similar genetic features, such as HLA DQ2 and DQ8, which are common in both AD^5^ and CD.^6^

Our index case represents the first-ever case of a pediatric patient with confirmed celiac disease and Addison’s disease with spontaneous recovery of adrenal functions. Smans et al. described a patient who was able to discontinue treatment after seven years but had a continued subnormal response to Synacthen tests.^7^ Recently, a second case was documented with biochemical Addison’s disease (peak cortisol 175 nmol/l) appearing during pregnancy and worsened by concomitant use of inhaled steroids and enzyme-inducing medications.^8^ During follow-up, the patient’s response to Synacthen varied, necessitating intermittent glucocorticoid replacement medication. Our case showed aberrant biochemistry at diagnosis (basal cortisol (8 a.m.) 45.79 nmol/L and ACTH 1250 pg/ml) and normal biochemistry after 1.5 years of stopping treatment (peak cortisol 500 nmol/L at 30 minutes post-synacthen). Her ACTH is still elevated, whereas serum electrolytes, renin, and cortisol responses to Synacthen are normal till date.

Potential explanations for the index case’s recovery of adrenal function could be an intense gluten-free diet has the potential to significantly lower autoimmune activity. This alteration in diet lowers the inflammatory response and may lessen the generation of autoantibodies linked to Addison’s disease and celiac disease. A gluten-free diet may cause the immune system to rebalance. Additionally, this may lessen the autoimmune attack on the adrenal glands.^9^ If left untreated, celiac disease frequently results in malabsorption of vital nutrients, including those that are critical for adrenal function, like magnesium, zinc, vitamins B12 and vitamin D. Eating a gluten-free diet promotes nutrient absorption by healing the gut lining. An improved diet may aid in the healing of the adrenal glands and the general functioning of the endocrine system.^2^ The intricate network that controls cortisol production is known as the hypothalamic-pituitary-adrenal (HPA) axis. Partial restoration of adrenal function may be possible if systemic health is enhanced and autoimmune activity is decreased. These factors may also favorably impact HPA axis control.^3^

The study done by Miconi F et al. revealed that a gluten-free diet had a pivotal therapeutic role in the resolution of Addison’s disease by possibly correcting nutrient deficiency that further helped in the restoration of adrenal functions.^2^ Cosnes et al. found similar results.^10^ They discovered that patients who complied had a lower cumulative risk of developing more autoimmune disorders over the course of ten years (6% vs. 15.6%; p = 0.02). Outcomes are predicted by age or gluten-free diet; we still do not know the exact answer.

These cases demonstrate that CD can be associated with AD in children, and a gluten-free diet appears to improve the course of AD. Indeed, further studies are required to ascertain if a gluten-free diet could be beneficial for coeliac patients diagnosed with Addison’s disease. This could potentially open new avenues for treatment and management of the disease.

### Learning Points:

- Recovery from Addison’s disease is rare, but possible.
- New reports of adrenal axis recovery may impact future counselling for Addison’s patients.

### Consent Statement:

Informed consent was obtained from parents for the publication of this data.

## CONCLUSION

This is the first case in children with clinical remission of autoimmune Addison’s disease. We suggest re-assessing the adrenal axis by synacthen test in children and adolescents with Addison disease, especially those whose hydrocortisone doses are gradually decreasing, to check for remission. Presently, there are no predictive models for patient recovery rates, necessitating consistent monitoring of individuals with ongoing endocrine disorders.

### Authors’ Contribution:

**KA:** Conceived, designed & editing of manuscript, is responsible for integrity of case report.

**SSH:** Did manuscript writing.

**SA:** Did review and drafting of manuscript.

All authors have read the final version of the manuscript and are accountable for the integrity of the study.
